# Evaluation of a micro/nanofluidic chip platform for diagnosis of central nervous system infections: a multi-center prospective study

**DOI:** 10.1038/s41598-020-58670-8

**Published:** 2020-01-31

**Authors:** Guanghui Zheng, Yan Zhang, Lina Zhang, Lingye Qian, Yumeng Cai, Hong Lv, Xixiong Kang, Dawen Guo, Xiaoming Wang, Jing Huang, Zhixian Gao, Xiuru Guan, Guojun Zhang

**Affiliations:** 1 Department of Clinical Diagnosis, Laboratory of Beijing Tiantan Hospital and Capital Medical University, Beijing, China; 2 National Engineering Research Centre for Beijing Biochip Technology, Beijing, China; 3 Daqing Oilfield General hospital clinical laboratory, Daqing, China; 4 Laboratory Diagnosis Department of the Affliated Hospital of Harbin Medical University, Harbin, China; 5 Department of Clinical Diagnosis, Laboratory of the First Hospital of Jilin University, Changchun, China; 6 Department of Neurosurgery of Beijing Tiantan Hospital and Capital Medical University, Beijing, China

**Keywords:** Meningitis, Biomedical engineering

## Abstract

Central nervous system infection (CNSI) is a significant type of infection that plagues the fields of neurology and neurosurgical science. Prompt and accurate diagnosis of CNSI is a major challenge in clinical and laboratory assessments; however, developing new methods may help improve diagnostic protocols. This study evaluated the second-generation micro/nanofluidic chip platform (MNCP-II), which overcomes the difficulties of diagnosing bacterial and fungal infections in the CNS. The MNCP-II is simple to operate, and can identify 44 genus or species targets and 35 genetic resistance determinants in 50 minutes. To evaluate the diagnostic accuracy of the second-generation micro/nanofluidic chip platform for CNSI in a multicenter study. The limit of detection (LOD) using the second-generation micro/nanofluidic chip platform was first determined using six different microbial standards. A total of 180 bacterium/fungi-containing cerebrospinal fluid (CSF) cultures and 26 CSF samples collected from CNSI patients with negative microbial cultures were evaluated using the MNCP-II platform for the identification of microorganism and determinants of genetic resistance. The results were compared to those obtained with conventional identification and antimicrobial susceptibility testing methods. The LOD of the various microbes tested with the MNCP-II was found to be in the range of 250–500 copies of DNA. For the 180 CSF microbe-positive cultures, the concordance rate between the platform and the conventional identification method was 90.00%; eight species attained 100% consistency. In the detection of 9 kinds of antibiotic resistance genes, including carbapenemases, ESBLs, aminoglycoside, vancomycin-related genes, and *mecA*, concordance rates with the conventional antimicrobial susceptibility testing methods exceeded 80.00%. For carbapenemases and ESBLs-related genes, both the sensitivity and positive predictive values of the platform tests were high (>90.0%) and could fully meet the requirements of clinical diagnosis. MNCP-II is a very effective molecular detection platform that can assist in the diagnosis of CNSI and can significantly improve diagnostic efficiency.

## Introduction

Central nervous system infection (CNSI) is a significant type of infection that currently plagues the fields of neurology and neurosurgery^1^. The primary symptoms of CNSI are meningitis and encephalitis syndrome. Common pathogens that cause CNSI include various species of bacteria, fungi, and viruses^2^, and some insects have been identified as vectors for CNSI-associated pathogens^3,4^. Of them, bacteria and fungi are the most significant CNSI-related pathogens, which seriously affect the health of neurology and neurosurgical patients with high morbidity and mortality^5,6^. By virtue of the existence of the blood-brain barrier, CNSI is difficult to treat with antibiotics. The ineffectiveness of antibiotics can result in delayed treatment, which can jeopardize the patient’s life. Consequently, prompt and accurate diagnosis of CNSI is a major challenge in clinical and laboratory evaluations.

There are currently several prompt and accurate diagnostic methods for CNSI in the clinical laboratory, such as multiplex PCR, high-throughput sequencing, Matrix-Assisted Laser Desorption/Ionization Time-of-Flight Mass Spectrometry (MALDI-TOF MS), microfluidic chip platform (MNCP), etc. However, most of these approaches have shortcomings; multiplex PCR is limited due to high rates of false positives in the detection procedure^7^, and high-throughput sequencing is expensive and may produce high rates of false positives, which ultimately limits its clinical application^8^. Furthermore, MALDI-TOF MS can be used for microbial identification, but this approach has its own technical limitations, MALDI-TOF MS requires bacterial colonies for identification, which will increase the time-costing of sub-culture into solid medium. Similar proteomics makes it difficult to distinguish bacteria, for example, when identifying *Shigella* and *Escherichia coli*^9^. Refined criteria are needed to distinguish closely related species and differentiate them from the next best taxon match^10^. In addition, it cannot perform antimicrobial susceptibility tests (AST)^11^. The MNCP is based on Loop-Mediated Isothermal Amplification (LAMP) technology that can solve the diagnosis issue of bacterial and fungal CNSI^12^. LAMP is different from PCR, mainly in two aspects: firstly, LAMP is amplified by isothermal temperature, usually 60–65 °C, in this study we used 65 °C. Secondly, a set of 4–6 primers of LAMP are designed for target DNA sequence, which are composed of 4 main primers called forward inner primer (FIP), backward inner primer (BIP), 2 outer primers (F3 and B3). In addition to the main primer, there are 0–2 loop primers. The special primers design for LAMP greatly improves its sensitivity^13^. In addition, LAMP and RT-LAMP has been widely used in pathogen detection field for its excellent specificity^14^. It can simultaneously detect multiple microbial indicators and antibiotic resistance genes without mutual interference. The microfluidic disc contains two main parts: the infusing channels (shown as curvy lines) and 48 reaction chambers (shown as dots). Primers are pre-pipetted into different reaction wells on the chip. Then a LAMP-compatible cover is used to seal the chip. During use, DNA template is pre-mixed with LAMP master mix and then pipetted into the chip through the loading inlet. The reaction mixture will fill the channel automatically and after centrifugation, it will be evenly centrifuged into different chambers and thoroughly mixed with primers. Both the chip’s inlet and outlet will be sealed with adhesive tape before placing the chip on an isothermal amplification detection device. The amplification process is monitored in real time and test results will be given at the end of the procedure. Targets with a significant increase in fluorescence signal in less than 50 minutes (tp < 50) and show a standard S curve are marked positive. In contrast with conventional identification and AST methods that may take up to 48 hours to obtain results, MNCP detection of microbial identification and antibiotic resistance genes can be achieved in 50 minutes, greatly improving the diagnosis efficiency of patients with CNSI and saving patient costs.

**Figure 1:**
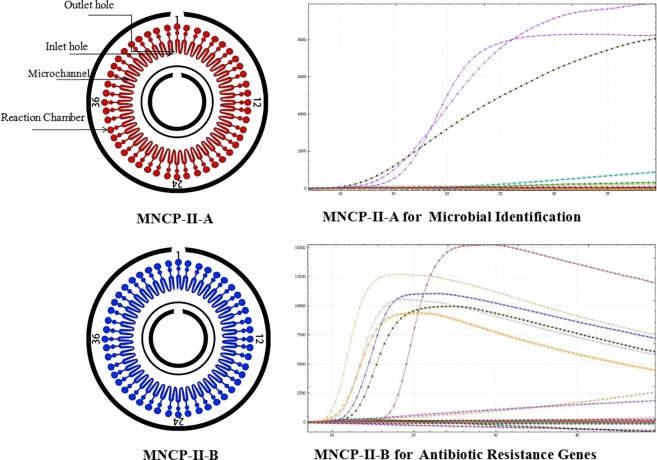
Schematic diagram of MNCP-II detection (The figure was created by Microsoft Office Powerpoint 365 and Adobe Photoshop CS 6, URL link “https://products.office.com/zh-cn/powerpoint” and “https://www.adobe.com/cn/products/cs6/photoshop.html”).

In 2017, we developed the first-generation MNCP (MNCP-I) to diagnosis post-neurosurgical bacterial infection. MNCP-I contains 10 bacterial identification parameters including *Klebsiella pneumonia, Pseudomonas aeruginosa, and Staphylococcus aureus*, and 13 antibiotic resistance gene parameters such as *bla*_*KPC*_, *bla*_*OXA-66*_, and *mecA*. The results showed that compared with the conventional method, MNCP-I had a concordance rate of 94% for diagnosis of post-neurosurgical bacterial infections. Thus, the MNCP-I showed promise in addressing issues caused by post-neurosurgical bacterial infection^12^, but post-neurosurgical infection accounts for only a fraction of CNSI incidence: the spontaneous causal pathogens of CNSI, such as *Streptococcus pneumonia* and *Haemophilus influenzae*, were not included in parameter sequence of MNCP-I. In addition, only 13 antibiotic resistance genes were selected in MNCP-I, and certain major resistance genes, such as the carbapenemase-related gene *bla*_*NDM-1*_ and porin-relevant gene *Ompk35*, were not included^15,16^. Despite the high concordance rate, sensitivity and specificity for β-lactam antibiotics, the MNCP-I still lacks robust clinical application. MNCP-I does not evaluate the diagnostic ability of MNCP-I for suspected infection and does not measure the different species’s limit of detection (LOD) of CNSI. To solve the problem of CNSI more systematically, we have redesigned the second-generation MNCP (MNCP-II). The MNCP-II still employs LAMP technology, but unlike the MNCP-I with its microbial and antibiotic resistance gene sequences configured on the same disk chip, MNCP-II is a parallel experiment of two chips: chip A is used for microbial identification, and chip B is used for antibiotic resistance gene screening (shown in Fig. 1). Thus, with the MNCP-II, more parameters can be obtained, which increases the extensiveness of clinical application significantly.

The aim of this study was to evaluate and validate the diagnostic value of MNCP-II in CNSI patients. We rescreened potential primers from species associated with CNSI and designed primer sequences to select potential resistance genes for these pathogens. A total of 44 bacterial and fungal parameters and 35 antibiotic resistance gene parameters were embedded into the MNCP-II-A and MNCP-II-B system. Meanwhile, considering the regional differences of microbes, we conducted a multicenter validation and selected 206 CNSI cases of four neurological centers in northern China. Diagnostic values of MNCP-II in CNSI were fully evaluated in this study. CSF and data collections and data were adhered to the Declaration of Helsinki and were approved by Beijing Tiantan Hospital Research Ethics Committee (Permission Numbers: KY 2019-095-03).

## Methods

### Design of the MNCP-II

Forty-four microorganisms were chosen as targets for the MNCP-II-A, including Gram-positive and Gram-negative bacteria, and various fungi (yeast, *Cryptococcus*, mold, etc.). For each of the microbes, 6 pairs of primers were selected. A total of 35 antibiotic resistance genes, including β-lactams, aminoglycosides, macrolides, quinolone, tetracycline and vancomycin resistance genes were chosen as the target MNCP-II-B sequences. By employing different primer sets, parallel detection of microbial and antibiotic resistance genes was possible. The specific parameters are shown in the supplementary material. In addition, pretreatment of the test, nucleic acid extraction, preparation of the isothermal amplification reaction solution and detection using the MNCP-II were the same as previously described for the MNCP-I^12^.

### Measurement limit of detection (LOD) of MNCP-II

*Acinetobacter baumannii* (ATCC 19606), *Staphylococcus aureus* (ATCC 25923), *Candida albicans* (ATCC 14053), *Streptococcus agalactiae* (ATCC 13813), *Klebsiella pneumoniae* (ATCC 10031) and *Enterococcus faecium* (ATCC 27336), were chosen as the six candidate microbe targets used in the determination of the LOD of MNCP-II. The procedure of the LOD was as follows: fresh colonies of the six target microbes were initially suspended in 1 ml of sterile water, and microbial suspensions having a turbidity of 0.5 McFarland were prepared (approximately 10^8^ colony-forming unit, CFU/ml)^17^. The microbe concentration gradient was obtained by a double or tenfold dilution method, and nucleic acid extraction was simultaneously performed to determine copies of the nucleic acid.

### Evaluation of the MNCP-II in CNSI patients

#### Identification of bacteria/fungus of MNCP-II

**Figure 2:**
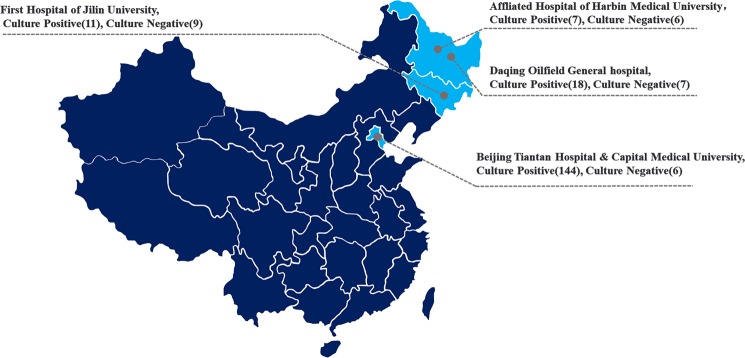
Location and distribution of the four centers enrolled in the MNCP-II evaluation trial (The figure was created by Microsoft Office Powerpoint 365, URL link “https://products.office.com/zh-cn/powerpoint”).

**Figure 3:**
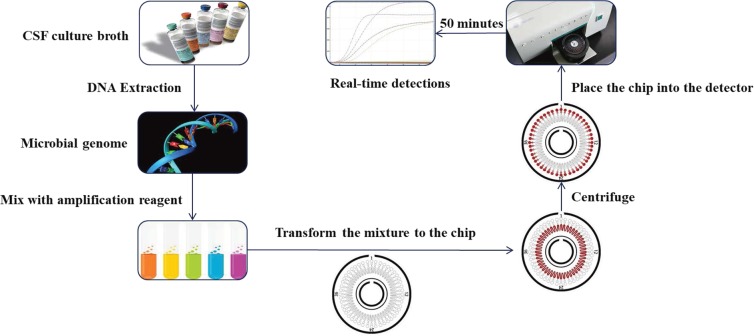
Flow chart of the MNCP-II test of a cerebrospinal fluid culture broth. (The figure was created by Microsoft Office Powerpoint 365 and Adobe Photoshop CS 6, URL link “https://products.office.com/zh-cn/powerpoint” and “https://www.adobe.com/cn/products/cs6/photoshop.html”).

A total of 206 patients with CNSI from 4 neurological centers in northern China were enrolled in this study for MNCP-II evaluation (Fig. 2). Of the total participants, 180 patients tested positive for cerebrospinal fluid (CSF) microbes by conventional culture methods, and 26 patients tested negative. Distributions of the patients are shown in Fig. 2: Beijing Tiantan Hospital & Capital Medical University (144 positive, 6 negative), First Hospital of Jilin University (11 positive, 9 negative), Daqing Oilfield General Hospital (18 positive, 7 negative), and Affiliated Hospital of Harbin Medical University (7 positive, 4 negative). Flow chart of the MNCP-II testing is shown in Fig. 3. Microbial identification and antimicrobial susceptibility testing were performed according to standard procedures. Briefly, 1–3 ml CSF was injected into bacterial/fungal culture bottles for automated 37 °C culture, and when it turns into culture positive (the automatic culture instrument will alarm), an aliquot of CSF broth was plated onto Columbia blood agar and incubated for 18–24 h in a 5% CO_2_ incubator. Colonies were then chosen for identification and antimicrobial susceptibility test.

The diagnosis of CNSI is currently made according to the standards proposed by the Center for Disease Control and Prevention (CDC)^18^. These standards include testing positive for microbial CSF culture or Gram stain; patient having at least 1 of the following symptoms with no other recognized cause: fever (>38 °C), headache, stiff neck, meningeal signs, cranial nerve signs, or irritability; and positive CSF examination: CSF leucocyte count ≥100/mm^3^ and CSF glucose <2.5 mmol/L or ratio of CSF glucose to blood glucose <0.4.

For the 180 CSF microbial culture-positive specimens, we verified the accuracy of microbial identification of MNCP-II-A through the comparison with the conventional methods (biochemical reaction or MALDI-TOF MS). All of the isolates were initially tested using a VITEK-2 Compact or VITEK-MS (bioMérieux, Marcy l’Etoile, France) to achieve microbial identification. Then, the concordance rate of the two procedures was estimated. When the two above methods were inconsistent, Sanger sequencing was employed for further verification. Additionally, the MNCP-II-A test was performed on 26 CSF microbial culture-negative specimens, and the specimens were subjected to Sanger sequencing to compare the consistency of the test results.

#### AST of bacteria of MNCP-II

ASTs of all microbes isolated from the CSF positive broth was performed in the clinical diagnosis departments of each of the four neurological centers by applying a standardized protocol. All of the isolates were initially tested with an automated VITEK-2 Compact AST system (bioMérieux, Marcy l’Etoile, France). In addition, minimum inhibitory concentrations (MICs) for all microbes were determined by broth micro-dilution using VITEK AST-GN and AST-GP cards in a VITEK-2 Compact instrument. Clinical and Laboratory Standards Institute breakpoint values (CLSI 2018, M100-S23) were applied^12^.

We compared the most commonly used antibiotics in neurological and neurosurgical patients related to antibiotic resistance genes and evaluated the clinical performance of MNCP-II-B in the diagnosis of CNSI. The diagnostic parameters, such as sensitivity, specificity, positive predictive values (PPVs), negative predictive values (NPVs), positive likelihood ratio (PLR), negative likelihood ratio (NLR), and overall concordance rate of MNCP-II-B with conventional methods were determined.

### Ethical approval

This study was approved was approved by the Beijing Tiantan Hospital Research Ethics Committee (Permission Numbers: KY 2019-095-03).

### Informed consent

Informed consent was obtained from all individual participants included in the study.

## Results

### LOD of MNCP-II

The LOD of the MNCP-II using the six standard microbes are shown in Table 1. The LOD of the six microbes range between 10^3^ CFU/ml and 10^6^ CFU/ml in concentration, and the minimum quantity of nucleic acid copies required for detection range between 250–500 copies.

**Table 1:** LOD of the six standard microbes using the MNCP-II.

Isolates	Microorganism	Concentration of Isolates	Nucleic acid Copies
*ATCC 14053*	*Candida albicans*	1*10^3^ CFU/ml	250
*ATCC 25923*	*Staphylococcus aureus*	1*10^6^ CFU/ml	500
*ATCC 10031*	*Klebsiella pneumoniae*	1*10^6^ CFU/ml	500
*ATCC 19606*	*Acinetobacter baumannii*	1*10^6^ CFU/ml	500
*ATCC 27336*	*Enterococcus faecium*	1*10^3^ CFU/ml	250
*ATCC 13813*	*Streptococcus agalactiae*	1*10^5^ CFU/ml	250

### Evaluation of the MNCP-II identification ability

The distribution of the 180 microorganisms isolated from CSF cultures during MALDI-TOF MS and conventional biochemical tests is shown in Table-2. Of them, 55 are Gram-positive bacteria, including *Streptococcus agalactiae, Staphylococcus aureus, Streptococcus pneumonia, Enterococcus faecalis, Staphylococcus epidermidis, Enterococcus faecium, Staphylococcus aureus, Staphylococcus hominis, and Staphylococcus haemolyticus*. There were also 119 Gram negative bacteria, including *Acinetobacter baumannii, Klebsiella pneumonia, Pseudomonas aeruginosa, Escherichia coli, Enterobacter aerogenes, Haemophilus influenza, Enterobacter cloacae*, and *Stenotrophomonas maltophilia*, and 5 isolates fungi, including *Candida albicans* and *Candida glabrata*. The performance of the MNCP-II-A for the identification of bacteria/fungus from CSF-positive cultures is summarized in Table 2.

**Table 2:** MNCP assay bacterial identification and concordance rate with the conventional method and Sanger sequencing.

Microorganism	Sanger sequencing	Conventional methods	Error	No-detected	MNCP-II	Concordance rate
Gram Positive Bacteria	55	55	5	1	49	89.09%
*Enterococcus faecalis*	5	5	1	0	4	80.00%
*Enterococcus faecium*	4	4	1	0	3	75.00%
*Staphylococcus aureus*	9	9	1	0	8	88.89%
*Staphylococcus capitis*	4	4	1	0	3	75.00%
*Staphylococcus epidermidis*	14	14	0	0	14	100.00%
*Staphylococcus haemolyticus*	1	1	0	0	1	100.00%
*Staphylococcus hominis*	6	6	0	0	6	100.00%
*Streptococcus agalactiae*	8	8	0	1	7	87.50%
*Streptococcus pneumoniae*	4	4	1	0	3	75.00%
Gram Negative Bacteria	119	119	6	5	108	90.76%
*Acinetobacter baumannii*	42	42	2	2	38	90.48%
*Enterobacter aerogenes*	3	3	0	0	3	100.00%
*Enterobacter cloacae*	5	5	1	0	4	80.00%
*Escherichia coli*	11	11	1	2	8	72.73%
*Haemophilus influenzae*	2	2	0	0	2	100.00%
*Klebsiella pneumoniae*	29	29	2	1	26	89.66%
*Pseudomonas aeruginosa*	26	26	0	0	26	100.00%
*Stenotrophomonas maltophilia*	1	1	0	0	1	100.00%
Fungus	5	5	0	1	4	80.00%
*Candida albicans*	3	3	0	1	2	66.67%
*Candida glabrata*	2	2	0	0	2	100.00%
Total	180	180	11	7	162	90.00%

All bacteria/fungi isolated from the CNSI patients’ CSF were identified by conventional methods, and Sanger sequencing was performed when the identification by conventional methods and MNCP-II-A were different. The results showed that Sanger sequencing was totally identical to MALDI-TOF MS detection. Compared with MALDI-TOF MS and biochemical reactions, MNCP-II-A has an accuracy of 90.00% for microbial identification. Among the groups, Gram-negative bacteria were most-accurately identified 90.76%, followed by Gram-positive bacteria (89.09%), and fungi (80.00%). For 8 microbial species, (*Staphylococcus epidermidis, Staphylococcus haemolyticus, Staphylococcus hominis, Enterobacter aerogenes, Haemophilus influenza, Pseudomonas aeruginosa, Stenotrophomonas maltophilia*, and *Candida glabrata*), identification accuracy was 100.00%. For the highest composition proportion in CNSI, *Staphylococcus epidermidis* (100.00%)*, Staphylococcus aureus* (88.89%)*, Acinetobacter baumannii* (90.48%)*, Klebsiella pneumonia* (89.66%), and *Pseudomonas aeruginosa* (100.00%), the identification accuracy exceeded 85.00%. The identification concordance rates of *Escherichia coli* and *Candida albicans* were low, only 72.73% and 66.67%, respectively.

MNCP-II was performed on cerebrospinal fluid culture broths from 26 CNSI patients testing negative for microbial infection via culture methods. The results showed that only 4 of the 26 cases were detectable by MNCP-II, and only 2 of those cases were verified by Sanger sequencing. The diagnostic efficiency for suspected infection was 8.33%(2/24).

### Evaluation of the MNCP-II AST predict ability

We evaluated MNCP-II-B on the resistance genes of Gram-positive and Gram-negative bacteria isolated from the CNSI patients in the four neurology centers previously listed. The gene distribution analysis of the resistance bacteria is shown in Table 3. The most common genes related to carbapenem resistance were *bla*_*KPC*_ (62.50%, 50/80), *bla*_*OXA-23*_ (48.75%, 39/80), and *bla*_*OXA-66*_ (42.50%, 34/80). The most common genes associated with ESBLs were *bla*_*TEM*_ (69.88%, 58/83), *bla*_*SHV*_ (67.47%, 56/83), and *bla*_*CTX-M-9*_ (39.76%, 33/83). The most common genes related to aminoglycosides, vancomycin, and macrolide are *aadA1*, *Van-A*, and *ermB*, with composition ratios of 96.55% (28/29), 70.00% (7/10), and 80.95% (17/21), respectively. *mecA* is the most frequent gene of oxacillin, with a composition ratio of 83.33% (15/18).

**Table 3:** Types and constituent ratios of resistance genes in microorganisms in CSF positive culture.

Phenotype (Resistance numbers)	Genes	Percentage
Carbapenem (80)	*bla*_*KPC*_	62.50%
	*bla*_*OXA-23*_	48.75%
	*bla*_*OXA-66*_	42.50%
	*bla*_*OXA-1*_	8.75%
	*bla*_*IMP*_	3.75%
	*bla*_*OXA-10*_	3.75%
	*bla*_*OXA-58*_	1.25%
	*bla*_*NDM*_	1.25%
ESBLs (83)	*bla*_*TEM*_	69.88%
	*bla*_*SHV*_	67.47%
	*bla*_*CTX-M-9*_	39.76%
	*bla*_*CTX-M-1*_	10.84%
Aminoglycosides (29)	*aadA1*	96.55%
	*aacC1*	3.45%
Vancomycin (10)	*Van-A*	70.00%
	*Van-B*	30.00%
Macrolide (21)	*ermB*	80.95%
	*mefA*	19.05%
Oxacillin (18)	*mecA*	83.33%

AST was performed by conventional methods using the VITEK-2 Compact instrument, as described previously. For Gram-negative bacteria, we chose three genes for comparison: Carbapenem, ESBLs and aminoglycoside-related genes, and the corresponding antibiotics were meropenem, ceftazidime plus clavulanic acid and amikacin, respectively. For Gram-positive bacteria, we also selected three types of genes, *mecA*, *Van*, and macrolide-related genes (*ermB* and *mefA*), and the corresponding antibiotics were oxacillin, vancomycin and erythromycin, respectively. The measured sensitivity, specificity, PPV, NPV, likelihood ratio and coincidence rate of MNCP-II-B were calculated, and the results are shown in Table 4.

**Table 4:** Sensitivity specificity, PPV, NPV, PLR, NLR, and concordance rate of microorganism detected by the MNCP-II-B.

MNCP-II-B	Sensitivity	Specificity	PPV	NPV	PLR	NLR	Concordance rate
Meropenem	93.02%	84.85%	94.12%	82.35%	6.14	0.08	90.76%
Ceftazidime plus clavulanic acid	92.22%	86.21%	95.40%	78.13%	6.69	0.09	90.76%
Amikacin	76.32%	83.95%	69.05%	88.31%	4.76	0.34	81.51%
Oxacillin	83.33%	90.32%	83.33%	90.32%	8.61	0.18	87.76%
Vancomycin	71.43%	88.57%	71.43%	88.57%	6.25	0.32	83.67%
Erythromycin	70.00%	84.21%	87.50%	64.00%	4.43	0.36	75.51%

Similar to MNCP-I, for Gram-negative bacteria, the concordance rate of phenotypes and genes of carbapenems and ESBLs were high (90.76%). Both the sensitivity and PPV of carbapenems and ESBLs were higher than 90%. The concordance rate of aminoglycosides to *aadA1* and *aacC1* was 81.5%, and the specificity and NPV were both higher than 80%. For Gram-positive bacteria, the concordance rate of oxacillin-resistant phenotype with *mecA* was 87.76% and the specificity and NPV were both higher than 90%. The concordance rate between vancomycin and *Van-A* and *Van-B* was 83.67%, and both specificity and NPV were higher than 80%. The concordance rate of erythromycin and *ermB* and *mefA* was 75.51%, slightly lower than 5 antibiotics above, but it had relatively high specificity and PPV (84.21% and 87.50%, respectively).

## Discussion

Currently, rapid diagnosis of CNSI caused by bacteria and fungi is a crucial challenge in the fields of neurology and neurosurgery. The literature reports that the incidence of post-neurosurgical CNSI is as high as 0.3%–25%^19^, and the proportion of spontaneous CNSI-related meningitis is also high, which can lead to serious health consequences and a significant increase in patient mortality^20^. At present, the clinical diagnosis of CNSI is mainly based on the combination of clinical symptoms and laboratory tests. Because the patient symptoms of microbial infection are similar to the clinical symptoms of stress reaction after neurosurgery, the routine methods based on microbial culture have always been required for accurate diagnosis; however, the combination of ineffective antibiotics and delayed diagnosis may jeopardize the health of the patient. Therefore, developing new molecular technologies or devices to diagnose CNSI is absolutely essential to improve current clinical practices.

In 2017, we developed the MNCP based on LAMP technology, which can integrate the gene sequences onto a fixed plastic disk. Compared with the conventional identification and AST methods, the MNCP detection exhibits better detection consistency and clinical applicability, and can produce rapid results in only 50 minutes. However, only 10 bacteria and 13 related genes were engineered into the MNCP, and only a single-center validation was applied. Both the applicability and practicability of the MNCP were insufficient for routine clinical use. Consequently, we built the MNCP-II, which was separated into A and B chips to detect pathogenic microorganisms and antibiotic resistance genes, respectively. Therefore, the MNCP-II expanded the detection range of the microbial and resistance genes. The LOD of the MNCP-II was assessed, and the identification and AST of the pathogens in four centers in northern China were selected for verification to obtain a universal detection platform of CNSI diagnosis.

Our research shows that MNCP-II, which can rapidly detect low concentrations of microorganisms, can be a valuable tool in diagnosis of CNSI. The results of the LOD of MNCP-II assessment show that microorganisms can be detected with stable sensitivity and specificity with as few as 250 nucleic acid copies (Table 1). However, for different microorganisms, sensitivity to MNCP-II detection is quite variable. For example, *Enterococcus faecium* only needs a concentration of 10^3^ CFU/ml to be detected, whereas *Staphylococcus aureus* and *Klebsiella pneumoniae* needs a concentration of 10^6^ CFU/ml to be reliably detected. The reason for these differences may be related to differences in DNA extraction efficiency between microbial species. This study also tested CSF specimens from patients that were diagnosed with CNSI, but tested negative for CSF microbial infection in culture, and the MNCP-II was unsuccessful in reliably detecting microorganisms in these samples. The reason for this low performance may be related to the fact that the concentration of microorganisms in culture is too low to reach the LOD of MNCP-II. In addition, broken gene fragments may be related to this phenomenon.

MNCP-II-A contains 44 microbial conservative area sequences, including spontaneous CNSI and post-neurosurgical CNSI-related microorganisms. The platform contains sequences of many microorganisms, including bacteria (Gram-positive and Gram-negative) and fungi (yeast, mold, *Cryptococcus*, etc.) that cover nearly all of the microorganisms known to cause CNSI. The results of CSF pathogen-positive cultures showed that MNCP-II-A is highly accurate in detecting CNSI-related pathogens, and the concordance rate compared with the conventional method is as high as 90.0%. Furthermore, accuracy of Gram-negative bacteria detection is slightly higher (90.76%) than Gram-positive bacteria (89.09%). As many as 8 types of pathogens were perfectly identified with the MNCP-II (concordance rate = 100%); the identification capacity of the MNCP-II for certain bacteria was significantly higher than MNCP-I. Accuracy of the identification of *Enterobacter cloacae* by MNCP-I was low (33% veracity), and after optimizing conditions such as prolonged detection time, the identification efficiency of 80.0% was obtained by MNCP-II. The problem of low identification efficiency for *Candida albicans* may be due to the small sample sizes. Nonetheless, in general, the detection of CNSI-associated pathogens by the MNCP-II-A is both quick and accurate, which is valuable for routine clinical application.

MNCP-II-B developed for bacterial resistance includes up to 35 common antibiotic resistance genes, and has higher gene coverage than the MNCP-I, which contains only 13 antibiotic resistance gene parameters. The MNCP-II-B not only contains carbapenemases, ESBLs, aminoglycoside, macrolide, vancomycin related genes, *mecA* and *Ompk35*, but also contains some antibiotic-related genes that are less clinically useful but are significant for antibiotic resistant bacteria, such as quinolone and tetracycline genes. We conducted a group study and found that the distribution of genes related to *Enterobacteriaceae* and non-fermentative bacteria is quite different. Among the carbapenem-related resistance genes, the most common antibiotic resistance gene of *Enterobacteriaceae* is *bla*_*KPC*_, but for non-fermentative bacteria, the most common gene is *bla*_*OXA-66*_. In terms of the effectiveness of application, the concordance rate of the carbapenem-related resistance gene and meropenem was found to be relatively high (90.76%), and the sensitivity and PPV were also higher than 90%. Both specificity and NPV were higher than 80%. Similar to carbapenem, MNCP-II-B had a concordance rate of 90.76%, 92.22% sensitivity, 86.21% specificity, 95.40% PPV, and 78.13% NPV in the detection of ESBLs-related genes. The concordance rate of aminoglycoside antibiotics and antibiotic resistance genes was 81.51%, and the specificity and NPV were higher (83.95% and 88.31%, respectively); however, the sensitivity rate (76.32%) and PPV (69.05%) were much lower. A possible reason for the lower sensitivity and PPV could be that the low quantity of aminoglycoside-relevant genes cannot fully cover the resistance gene. For Gram-positive bacteria, *mecA* is the most common antibiotic resistant gene of MRSA/MRCoNS. Test results show that the concordance rate is 87.76%; sensitivity, specificity, PPV and NPV were all higher than 80%^21,22^. Similar with the literature reports^23,24^, vancomycin-related genes (*Van-A*, and *Van-B*) are the most popular genes in *Enterococcus*; the concordance rate, specificity and NPV of vancomycin-related genes and phenotypes were higher than 80%, while the sensitivity rate and PPV were higher than 70%. The initial detection of *Enterococci* infection also has certain clinical significance. In addition, the consistency of erythromycin with related genes is insufficient, which may be similar to aminoglycoside antibiotics, and gene coverage is low.

We have demonstrated the utility of the MNCP-II in detecting low concentrations of microorganisms associated with CNSI, but the MNCP-II still has its shortcomings.. In addition to the data discussed in the results, we also embedded some quinolone and tetracycline-related genes, including *tetC*, *tetW*, *tetQ*, *qnrA*, and *qnrS*, which are reported in the literature^25^; however, their consistency in producing results is poor. There may be two reasons for this: (1) antibiotic-related resistance genes have been mutated, (2) the bacteria selected in this study have fewer genes related to these two antibiotics, resulting in poor generalization. Therefore, in future research, a wider range of bacteria should be selected for verification and the antibiotic resistance gene data should be obtained by sequencing. In terms of application, the LOD of MNCP-II is still maintained at 10^3^ CFU/ml, and the sensitivity is lower than that of digital PCR and high-throughput sequencing. It may have certain application value for the diagnosis of severe infection of spontaneous CNSI (with high microbial density); however, for most post-neurosurgical CNSI, the application is relatively limited and only severe CSF infections in critically ill patients can be directly detected. We expect that the sensitivity can be improved by optimizing the conditions of a third-generation MNCP-III system.

In summary, MNCP-II is a very effective molecular detection platform that can assist in the diagnosis of CNSI and can significantly improve the diagnostic efficiency and shorten the detection time in comparison to current laboratory methods. It is a novel and effective tool in clinical diagnosis, and has potential to greatly improve the detection of neurological and neurosurgical infections.

## Supplementary information

supplementary material.
