# Supplementary material for: Evaluation of a micro/nanofluidic chip platform for diagnosis of central nervous system infections: a multi-center prospective study
Source: Sci Rep. 2020 Jan 31;10:1568. doi: 10.1038/s41598-020-58670-8 (PMC6994612; doi:10.1038/s41598-020-58670-8)
Supplement: Supplementary file 1 — supplementary material. [file 41598_2020_58670_MOESM1_ESM.pdf]

Evaluation of a micro/nanofluidic chip platform for diagnosis central nervous system infections: a multi-center prospective study

Guanghui Zheng[a]#, Yan Zhang[b]#, Lina Zhang[c]#, Lingye Qian[a], Yumeng,Cai[a], Hong, Lv[a], Xixiong, Kang[a], Dawen Guo[d], Xiaoming Wang[e], Jing Huang[e]*, Zhixian Gao[f]*, Xiuru Guan[d]*, Guojun Zhang[a]*

a Department of Clinical Diagnosis, Laboratory of Beijing Tiantan Hospital and Capital Medical University, Beijing, China

b National Engineering Research Centre for Beijing Biochip Technology, Beijing, China

c Daqing Oilfield General hospital clinical laboratory, Daqing, China

d Laboratory Diagnosis Department of the Affliated Hospital of Harbin Medical University, Harbin, China

e Department of Clinical Diagnosis, Laboratory of the First Hospital of Jilin University, Changchun, China

f Department of Neurosurgery of Beijing Tiantan Hospital and Capital Medical University, Beijing, China

Corresponding author:

Guojun Zhang*, Department of Clinical Diagnosis, Laboratory of Beijing Tiantan Hospital and Capital Medical University, Beijing, China, E-mail:zgjlunwen@163.com.

Jing Huang*, Department of Clinical Diagnosis, Laboratory of the First Hospital of Jilin University, Changchun, China, E-mail:jluhuangjing1@126.com

Zhixian Gao*, Department of Neurosurgery of Beijing Tiantan Hospital and Capital Medical University, Beijing, China E-mail:zhixian_g@163.com

Xiuru Guan*, Laboratory Diagnosis Department of the Affliated Hospital of Harbin Medical University, Harbin, China, E-mail:guanxiuru0451@163.com

Guanghui Zheng[#], Yan Zhang[#] and **Lina Zhang**[#] contributed equally to this study

Supplementary material-1: Identification parameters in MNCP-II-A

NO.	Microorganism	NO.	Microorganism
1	*Acinetobacter baumannii*	23	*Haemophilus influenzae*
2	*Aspergillus flavus;*	24	*Klebsiella pneumoniae*
3	*Aspergillus fumigatus*	25	*Legionella pneumophila*
4	*Aspergillus nidulans*	26	*Leptospira*
5	*Aspergillus niger*	27	*Mycobacterium tuberculosis complex*
6	*Aspergillus terre*	28	*Mycoplasma pneumoniae*
7	*Borrelia burgdorferi*	29	*Neisseria meningitidis*
8	*Brucella maltese*	30	*Penicillium marneffei*
9	*Candida albicans*	31	*Proteus mirabilis*
10	*Candida glabrata*	32	*Pseudomonas aeruginosa*
11	*Candida krusei*	33	*Squirrel staphylococcus*
12	*Candida parapsilosis*	34	*Staphylococcus aureus*
13	*Candida tropicalis*	35	*staphylococcus capitis*
14	*Chlamydia pneumoniae*	36	*Staphylococcus epidermidis*
15	*Clostridium perfringens*	37	*Staphylococcus haemolyticus*
16	*Cryptococcus Gatti*	38	*Staphylococcus hominis*
17	*Cryptococcus neoformans*	39	*Stenotrophomonas maltophilia*
18	*Enterobacter aerogenes*	40	*Streptococcus agalactiae*
19	*Enterobacter cloacae*	41	*Streptococcus mutans*
20	*Enterococcus faecalis*	42	*Streptococcus pneumoniae*
21	*Enterococcus faecium*	43	*Streptococcus pyogenes*
22	*Escherichia coli*	44	*Treponema pallidum*

Supplementary material-2: Antibiotic resistance gene parameters in MNCP-II-B

NO	Resistance Gene	NO	Resistance Gene
1	*mecA*	19	*ermB*
2	*Van-A*	20	*mefA*
3	*Van-B*	21	$bla_{OXA\text{-}24}$
4	$bla_{OXA\text{-}23}$	22	$bla_{DHA\text{-}1}$
5	$bla_{OXA\text{-}48}$	23	*CMY*-2
6	$bla_{OXA\text{-}58}$	24	*ACT*
7	$bla_{OXA\text{-}66}$	25	*cfxa*
8	$bla_{GES\text{-}1}$	26	$bla_{OXA\text{-}1}$
9	$bla_{CTX\text{-}M\text{-}1}$	27	$bla_{OXA\text{-}10}$
10	$bla_{CTX\text{-}M\text{-}9}$	28	*tetC*
11	bla_{IMP}	29	*tetW*
12	bla_{VIM}	30	*tetQ*
13	bla_{KPC}	31	*qnrA*
14	bla_{NDM}	32	*qnrS*
15	bla_{TEM}	33	*ompK35*
16	bla_{SHV}	34	*arr*-2
17	*aadA1*	35	*mcr*-1
18	*aacC1*		
